# Partnering around cancer clinical trials (PACCT): study protocol for a randomized trial of a patient and physician communication intervention to increase minority accrual to prostate cancer clinical trials

**DOI:** 10.1186/s12885-017-3804-5

**Published:** 2017-12-02

**Authors:** Susan Eggly, Lauren M. Hamel, Elisabeth Heath, Mark A. Manning, Terrance L. Albrecht, Ellen Barton, Mark Wojda, Tanina Foster, Michael Carducci, Dina Lansey, Ting Wang, Rehab Abdallah, Narineh Abrahamian, Seongho Kim, Nicole Senft, Louis A. Penner

**Affiliations:** 1 Department of Oncology, Wayne State University/Karmanos Cancer Institute, 4100 John R, Detroit, MI 48201 USA; 2 Department of English, Wayne State University, 5057 Woodward Suite 9408, Detroit, MI 48202 USA; 3 Johns Hopkins School of Medicine/Sidney Kimmel Comprehensive Cancer Center, 1M59 Bunting –Blaustein Cancer Research Building, 1650 Orleans Street, Baltimore, MD 21287 USA; 4 Johns Hopkins School of Medicine/Sidney Kimmel Comprehensive Cancer Center, 550 North Broadway, 1003-G, Baltimore, MD 21205 USA

**Keywords:** Patient-physician communication, Health disparities, Prostate cancer, Clinical trials

## Abstract

**Background:**

Cancer clinical trials are essential for testing new treatments and represent state-of-the-art cancer treatment, but only a small percentage of patients ever enroll in a trial. Under-enrollment is an even greater problem among minorities, particularly African Americans, representing a racial/ethnic disparity in cancer care. One understudied cause is patient-physician communication, which is often of poor quality during clinical interactions between African-American patients and non-African-American physicians. Partnering Around Cancer Clinical Trials (PACCT) involves a transdisciplinary theoretical model proposing that patient and physician individual attitudes and beliefs and their interpersonal communication during racially discordant clinical interactions influence outcomes related to patients’ decisions to participate in a trial. The overall goal of the study is to test a multilevel intervention designed to increase rates at which African-American and White men with prostate cancer make an informed decision to participate in a clinical trial.

**Methods/design:**

Data collection will occur at two NCI-designated comprehensive cancer centers. Participants include physicians who treat men with prostate cancer and their African-American and White patients who are potentially eligible for a clinical trial. The study uses two distinct research designs to evaluate the effects of two behavioral interventions, one focused on patients and the other on physicians. The primary goal is to increase the number of patients who decide to enroll in a trial; secondary goals include increasing rates of physician trial offers, improving the quality of patient-physician communication during video recorded clinical interactions in which trials may be discussed, improving patients’ understanding of trials offered, and increasing the number of patients who actually enroll. Aims are to 1) determine the independent and combined effects of the two interventions on outcomes; 2) compare the effects of the interventions on African-American versus White men; and 3) examine the extent to which patient-physician communication mediates the effect of the interventions on the outcomes.

**Discussion:**

PACCT has the potential to identify ways to increase clinical trial rates in a diverse patient population. The research can also improve access to high quality clinical care for African American men bearing the disproportionate burden of disparities in prostate and other cancers.

**Trial registration:**

Clinical Trials.gov registration number: NCT02906241 (September 8, 2016).

## Background

Cancer clinical trials are essential for testing the safety and efficacy of promising treatments and translating new knowledge into tangible benefits for patients; they also represent state-of-the art treatment for individuals with cancer [1, 2]. However, only a small percentage of cancer patients ever enroll in a trial [3, 4]. Estimates of the proportion of trials that fail to meet scientific objectives because of insufficient accrual range from 22 to 50% [5, 6]. Low accrual jeopardizes researchers’ ability to assess the safety and effectiveness of new approaches to cancer care, wastes resources, and precludes follow-up studies [6, 7].

Despite NIH requirements to include minorities in clinical research, [8] under-enrollment is an even greater problem among minorities, particularly African Americans [4, 9–13]. Minority under-enrollment can limit the generalizability of findings to those racial/ethnic groups studied [10, 13, 14]. Further, given the National Academy of Science’s recommendation that every individual with cancer should have access to high quality clinical trials [2], minority under-enrollment represents a racial/ethnic disparity in cancer treatment that may lead to disparities in outcomes and survival [1, 15, 16].

Under-enrollment of African Americans and other minorities is often attributed to patients’ negative attitudes toward trials [17–19], but research suggests a more complicated picture [13, 20–23]. National and system factors, such as a lack of available trials, strict eligibility criteria, and competing demands on under-resourced hospitals also present significant barriers that likely have a disproportionate effect on minority enrollment [2, 9, 21, 24–27]. Several national, regional, and consortia efforts are addressing either patient or system factors [13, 22, 28, 29]. However, even when medical institutions have an adequate trial infrastructure and trials are available, physicians are often unwilling or unprepared to discuss trials with some patients, and patients are often mistrustful of physicians or of trials, especially racial/ethnic minority patients [27].

**Fig. 1:**
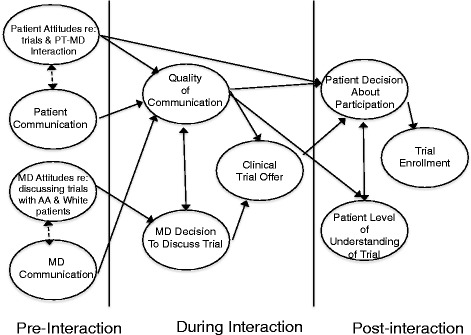
Conceptual Model

Partnering Around Cancer Clinical Trials (PACCT) is a behavioral intervention based on a conceptual model (Fig. 1) that translates theories from social psychology and communication science to address the critical need to increase minority participation in clinical trials. The conceptual model proposes that patient and physician individual attitudes and beliefs prior to a clinic visit and their interpersonal communication during the clinic visit interact to directly and indirectly influence outcomes related to patients’ decisions about trial participation. The conceptual model provides a theoretical framework for the intervention designed to improve rates of clinical trial participation among African-American and White men with prostate cancer. The following paragraphs describe the conceptual model.

As illustrated in Fig. 1, the quality of patient-physician communication during clinical interactions is considered the most central and proximal influence on patients’ decisions about participating in trials. We focus on communication for two reasons: first, because it is through these interpersonal processes among health care organizations, providers, patients, and families that health care is transacted [30, 31]; and second, because communication during clinical interactions with African-American patients and non-African-American physicians (i.e., racially discordant interactions) has been shown in our and others’ research to be lower in quality than in comparable clinical interactions with White patients [32–40]. This is particularly important because very few oncologists are African American, and thus oncology interactions for African-American patients are almost always racially discordant [41].

Our model suggests that patients’ and physicians’ individual attitudes and beliefs prior to a clinical interaction directly and indirectly affect the quality of communication during the interaction, and in turn, affect decisions that physicians make about discussing trials and that patients make about participating in the trial. These attitudes and beliefs include those that prior research shows may affect the quality of communication during clinical interactions in which trials are discussed. With regard to African-American patients, research shows that overall, members of this racial group are as likely as White patients to consent if they are offered a trial [18, 26, 42–44]. However, some African Americans hold race-related attitudes and beliefs that could directly and indirectly influence whether and how a physician discusses a trial with them and how they respond to these discussions [17, 45–47]. These attitudes, derived in great part from the legacy of racism and poorer health care for minorities in the U.S [48–50], include greater mistrust in medical institutions and physicians, higher suspicion about how healthcare systems treat African-American patients, and increased perceptions of having been the target of discrimination [51–59]. Our work and that of others show that these attitudes lower the quality of communication in interactions with African-American patients and their non-African-American physicians [36, 53, 59], and may, in part, explain why communication in these racially discordant interactions is often of lower quality compared to communication with White patients. With regard to physicians, research suggests that some physicians have negative feelings about African-American patients [60] and may believe they are poor candidates for clinical trials because of racial stereotypes that they are less educated, less trustworthy, or less compliant [61–64]. These attitudes, which are often implicit rather than explicit, could influence whether and how a physician discusses a trial, and could also result in physicians opting for less aggressive treatments for African American patients [65–67].

As also illustrated in Fig. 1, the conceptual model focuses on the quality of patient-physician communication during clinical interactions as a primary influence on patients’ decisions about participating and their understanding of the key aspects of the trial. In PACCT, we are primarily concerned with aspects of communication that may be affected by the topic of clinical trials, and that may vary with patient race. These aspects of communication include patient active participation in clinical interactions (e.g., asking questions, stating concerns) [68, 69], physician patient-centeredness (e.g., patient-centered communication, shared decision making) [31, 70], and the extent to which physicians discuss a trial and clearly explain key aspects of consent (e.g., purpose, risks, benefits of trial participation) [38, 71].

Based on the conceptual model, and consistent with recent calls to move beyond single-level interventions [72–74], PACCT will test two interventions: one focused on patients and the other on physicians. Independently and together, these interventions are designed to influence *patients’* attitudes about physicians and about trials; *physicians*’ attitudes about patients and about trials; and *patient-physician clinical interactions* in which trials may be discussed. The primary goal is to improve the rates at which men decide to participate in a prostate cancer clinical trial, based on high-quality communication with their physicians. Secondary goals are to improve rates at which physicians discuss and offer trials to eligible patients, the quality of patient-physician communication during interactions in which trials may be discussed, patients’ understanding of trials offered, and rates of actual accrual to clinical trials. More specifically, PACCT is designed to achieve the following *aims* and test the following *hypotheses*:

- Aim 1. Determine the effects of the patient- and physician-focused interventions on outcomes. The primary outcome is improved rates of patients’ decisions to enroll in a clinical trial; the secondary outcomes are physicians’ offers of a trial, the quality of patient-physician communication during clinical interactions, patients’ understanding of the trial offered, and patients’ actual enrollment in the trial.
  - Determine the effects of the patient-focused intervention on outcomes. **Hypothesis 1a**: Outcomes will be significantly improved in the patient intervention group, relative to a usual care group.
  - Determine the effects of physician-focused intervention on outcomes. **Hypothesis 1b**: Outcomes will be significantly improved for patients after the physician intervention, as compared to outcomes before the physician intervention.
  - Determine the combined effects of the two interventions on outcomes. **Hypothesis 1c**: There will be a significant multiplicative effect of the two interventions that yield improvements in primary and secondary outcomes over and above the independent effects of each intervention.
- Aim 2. Compare the effects of the interventions on outcomes for African American versus White men. **Hypothesis 2**: The effects of the two interventions will be significantly greater among African American than White men.
- Aim 3. Examine the extent to which patient-physician communication mediates the relationship between the intervention and outcomes. **Hypothesis 3**: The quality of communication will mediate the effects of the patient and physician intervention on trial offers, and, in turn, on patient understanding of trials offered and decisions to participate. Because the specific meditational variables to be tested will emerge from the analyses related to the first two hypotheses, this is an exploratory hypothesis.

## Methods/Design

### Study design

PACCT is a clinical trial involving two behavioral interventions, one focused on patients and the other on physicians, each evaluated with a distinct research design. The patient-focused intervention is evaluated with a between-subjects randomized controlled trial in which patients are randomized to an intervention or usual care group, and outcomes are compared between groups. The physician-focused intervention is evaluated with a within-subjects interrupted time series design in which physicians participate during a pre-intervention period (approximately 20 months) followed by the intervention (2 months), and then a post-intervention period (approximately 20 months). In order to assess change, the planned outcomes are assessed prior to and then following the intervention.

### Participants and setting

PACCT will be conducted at two National Cancer Institute-designated comprehensive cancer centers: Wayne State University/Karmanos Cancer Institute (WSU/KCI) in Detroit, Michigan, and John Hopkins Medicine/Sidney Kimmel Comprehensive Cancer Center (SKCCC) in Baltimore, Maryland. Physicians (medical oncologists, urologists, and radiation oncologists) are eligible to participate if they regularly treat patients with prostate cancer at one of the two research sites and can recruit patients to available trials. Adult patients are eligible to participate if they have a confirmed diagnosis of prostate cancer; self-identify as Black, African American, or White and non-Hispanic; have been seeing a participating oncologist for less than a year and expect to see the physician at least once in the following year; are able to read and write English well enough to understand the consent documents and respond to questionnaire; and are potentially eligible for a clinical trial within two years of consent.

### Procedures

#### Physicians

Up to 24 physicians will be recruited at the beginning of data collection, prior to patient recruitment. To recruit physicians, research staff will attend departmental meetings to explain the study, and then invite interested physicians to meet individually to answer questions and obtain consent. Physicians who consent will agree to complete baseline measures, to inform their eligible patients about this study during a regularly scheduled clinic visit, to allow video recording of selected patient visits, to complete a brief questionnaire after video recorded patient visits, and to participate in a training intervention in approximately two years. Physicians will continue their participation throughout the study period (approximately 4 years). Baseline measures (see Table 1) will include socio-demographic characteristics, attitudes toward trials and toward the patient-physician relationship, and widely-used assessments of explicit and implicit racial attitudes about African-American and White people. Post-interaction measures will assess physicians’ perceptions of patients and whether a trial was discussed. Physicians receive a $50 gift card for their participation in the study.

**Table 1:** Study measures

	Time 0 Consent	Time 1 1 week prior to clinic visit	Time 2: Clinic Visit	Time 3: Follow-up interview
Patient measures				
Socio-demographics (e.g, age, race/ethnicity, education, income)	X			
Date of prostate cancer diagnosis	X			
Economic burden [97]	X			
Health status [98]	X	X		
Health literacy [99, 100]	X			
Trust in the medical profession [101]	X			X
Group-based medical mistrust [102]	X			
Receptivity to discussing a clinical trial [103]	X			
Decisional control preferences [69, 104]		X		
Patient-Practitioner Orientation Scale [105]		X		
Self-efficacy with discussing trials		X		
Positive and Negative Affect Scale (PANAS) [106]	X		X	
Attitudes toward clinical trials		X		
Trust in a physician [101]		X		
Perceived racial/ethnic discrimination [107]		X		
Religiosity [108]		X		
Spirituality [109]		X		
Social support [110]		X		
Decisional control perceptions [111]			X	
Perceived physician patient-centeredness [112]			X	
Perceived active participation in the interaction [37]			X	
Perceived physician patient-centered communication [37]			X	
Presence of a trial discussion/offer			X	
Decision about participating in trial offered				X
Understanding of trial offered [113]				X
Perceptions of team [114]				X
Satisfaction with intervention (intervention arm only) [69]				X
Open-ended questions regarding trial offered				X
Physician Measures				
Socio demographic/professional characteristics (e.g., age, race/ethnicity, years in practice)	X			
Attitudes toward clinical trials [25, 115]	X			
Attitudes toward offering a clinical trial	X			
Decisional control preferences [69, 111]	X			
Patient-Practitioner Orientation Scale [105]	X			
Racial attitudes/symbolic racism [116]	X			
Implicit racial attitudes [117]	X			
Perceptions of patient [67]		X		
Presence of a trial discussion/offer		X		
Decisional control perceptions [111]		X		
Observer Ratings of Video Recorded Interactions				
Presence and quality of clinical trial discussion [38]			X	
Physician patient-centered communication [37]			X	
Patient active participation in interaction [37]			X	

#### Patients

**Fig. 2:**
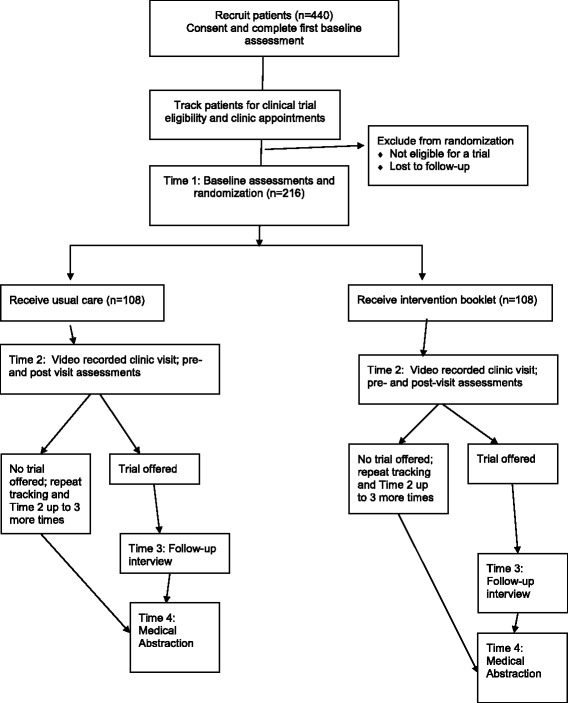
Flow Diagram of Patient Enrollment, Randomization, and Procedures

Patient procedures are illustrated in Fig. 2. Up to 440 patients will be recruited in two waves, the first half immediately following physician consent and the second half immediately following the physician intervention. Within each wave, equal numbers of African-American and White patients will be recruited. Up to 16 patients will be recruited per physician in each wave. Research staff will identify eligible patients who have an appointment with a participating physician. Physicians (or their designee) will inform these patients about the study. Research staff will meet with interested patients to explain the study, obtain consent, and have them complete a brief questionnaire (see Table 1). Patients will receive a $20 gift card at this time. Research staff will then track patients until they become potentially eligible for an available clinical trial and have a scheduled appointment with a participating physician. Patients who do not become eligible for a clinical trial during the study period will have no further contact with research staff. Patients who are found to be potentially eligible for a trial will be asked to participate in up to four more study sessions.

#### Time 1 (prior to clinic visit)

When research staff determines that a participating patient is potentially eligible for an available clinical trial and has an appointment with a participating physician, they will contact him approximately one week before the appointment, remind him about the study, and arrange to meet with him at a convenient time and place to complete a questionnaire (see Table 1). The research staff will NOT directly inform patients about their potential trial eligibility; if asked, they will encourage patients to discuss this with their physician. Once the questionnaire is completed, an automated computer program provided by Qualtrics^@^ will randomly assign the patient to either the usual care or intervention group (1:1). Intervention group patients will receive the intervention (i.e., booklet and instructions) at this time. All patients will receive a $20.00 gift card and be told that their next clinic visit may be video recorded.

#### Time 2 (clinic visit)

On the day of the clinic visit, research staff will meet with patients to remind them that the visit will be video recorded and to ask patients to complete brief questionnaires just prior to and following the visit (see Table 1). If family members or companions are present, they will be told about the study and asked for consent to be video recorded, but will not complete any questionnaires. Similarly, clinical staff who will be in the exam room during video recording will be asked for consent. Patients will receive a $10.00 gift card following this visit. Patients will be asked whether they were offered a clinical trial; if they were not, they will be told that they are still in the study and may be contacted again in the future. They will continue to be tracked for up to a total of four visits or until a trial is offered. If they still receive no offer after a fourth visit, they will no longer be tracked. If they are offered a trial, they will proceed to Time 3.

#### Time 3 (follow-up interview)

A week after the visit, research staff will contact patients (on the phone or in person as convenient to patients) who were offered a trial to conduct a brief interview (see Table 1). Patients will receive a $10.00 gift card at the end of this interview.

#### Time 4 (medical record review)

Research staff will examine patient medical records to identify potential covariates to be included in the analysis, such as patients’ disease status and co-morbidities. Staff will also determine whether patients completed procedures for trial enrollment and/or enrolled in a trial; and trial characteristics (e.g., difficulty, complexity).

### Interventions

#### Patient intervention

The patient-focused intervention includes both attitude and communication components and is in the form of a booklet. The first section, the attitude component, is based on the well-researched Common Ingroup Identity Model [75, 76]. Extensive research shows that establishing a sense of common identity or purpose between interaction participants increases cooperation and trust among members of different social groups. Briefly, the booklet tells patients that they and their physicians have equally important roles and need to work together as a team to provide the best care for the patient’s cancer. Research assistants will briefly review this section with patients randomized to the intervention group and ask them to place their initials at the bottom of the page to confirm their role as member of the patient-doctor team. The second section, the communication component, is a Question Prompt List (QPL), which includes instructions and a list of questions related to clinical trials. This communication tool has been used in several settings to encourage and assist patients to participate actively during medical visits [77–79]. Patients prepared with a QPL may be more likely to ask questions and state their concerns about trials and/or treatments, potentially enabling a shared decision making process. The QPL for PACCT was adapted from two existing QPLs. The first was a booklet developed in collaboration with patients, oncologists, and community members for use as an intervention in a study of African-American patients facing a discussion with an oncologist about chemotherapy [69, 80]. The second was a QPL developed specifically for use during interactions involving a discussion of clinical trials [81]. After patients have finished reading the “team” component of the booklet, research staff will tell patients that the list was developed by doctors and patients, and that patients might find it helpful during the clinic visit, especially if they discuss a clinical trial with their doctor. The research assistants will be trained NOT to answer questions nor discuss trials, but rather to encourage patients to ask questions during clinic visits. The intervention meetings will be audio-recorded to assess fidelity to the protocol.

#### Physician intervention

The physician-focused intervention, which begins about 20 months after the start of the overall study, includes two components: a communication and an attitude component. The communication component consists of a web-based training module whose objective is to improve physicians’ communication skills in general (e.g., patient-centeredness, shared decision making) and specific to discussing trials with patients (e.g., key aspects of consent). During the training, physicians will view a video that provides information about the importance of recruiting a diverse population of patients to cancer clinical trials, and reflect on communication skills that facilitate effective patient-centered communication and shared decision-making about trials. Training methods will include brief explanations and discussions and video illustrations.

The training is based on communication theory that suggest that in clinical communication, participants exchange both informational and relational messages [71]; the web-based training will include training in how to provide both. Skill-building in *informational communication* involves guidelines for discussing information patients need to make an informed decision about participating in a trial based on the International Ethical Guidelines for Biomedical Research Involving Human Subjects prepared by the Council for International Organizations of Medical Sciences (CIOMS) and the World Health Organization (WHO) (https://cioms.ch/wp-content/uploads/2017/01/WEB-CIOMS-EthicalGuidelines.pdf). [82] Skill-building in *relational communication* involves explanations and illustrations of communication strategies such as using organizing statements, eliciting questions and concerns (e.g., “Ask-Tell-Ask”), using lay language, assessing understanding by using the “teach-back” method, acknowledging and responding directly and empathically to questions and concerns, and using shared-decision making principles [82–87].

The attitude component of the intervention will take place after physicians complete the communication component, and is designed to increase the likelihood that physicians will discuss and offer trials to their patients. There are two elements of the attitude component: an attitude accessibility element and a situation-specific plan element. The attitude accessibility element is intended to make positive attitudes about the scientific and clinical benefits of offering a trial more accessible and salient to physicians. The situation-specific plan element is intended to further increase the probability that attitudes will be translated into actions. Together, both elements will be provided to physicians via a brief email a few days before *each visit* with a participating patient in the second wave (post physician intervention) who is potentially eligible for a clinical trial. The email will ask physicians to rate the clinical and scientific benefits of offering this patient a trial, and to indicate what they will do to prepare each patient for a discussion about trials.

#### Observational measures (see Table 1)

Trained raters will observe and rate video recorded visits. We will follow procedures used in our prior studies to train raters and ensure acceptable inter-rater reliability. Raters will determine whether a trial was discussed and/or offered and assess the quality of trial-related communication [38]; physician patient-centeredness [37], and patient active participation in the interaction [37].

### Sample size calculation/analyses

A randomized controlled trial will be used to evaluate the patient-focused intervention and a within-subjects design to evaluate the physician intervention. However, the outcomes of both interventions will be modeled at the patient level in a single multilevel model (MLM; i.e., patients nested within physicians). This model allows us to simultaneously examine the main effect of each intervention and multiplicative effects of having been exposed to both interventions. We will use binomial logistic models for binary outcomes (e.g., trial offer) and multinomial logistic regression for categorical outcomes (e.g., patients’ self-reported participation decision - “yes”, “no”, “undecided”). We will model other outcomes (specifically, patients’ perceptions of patient-centeredness, trust in physician, team perceptions, active participation, physician patient-centeredness, and patient understanding of informed consent) as continuous variables. We used the person-level multi-site/block trial design within Optimal Design to conduct power analyses because the unit of analysis is the patient-physician visit and data from these visits will likely be more similar within physicians than between physicians. The first power analysis is based on the 216 patients who are found to be eligible for a clinical trial and randomized to receive the intervention or usual care (See Fig. 2). We define the *primary outcome* of our study as patients’ decisions to enroll in a clinical trial. Aim 1 is to examine the extent to which the patient- and physician-focused interventions affect patients’ decisions to enroll, and thus we seek a sample size that gives us sufficient power to detect both the main effect of intervention and important interaction effects. We chose the power analysis in General Estimating Equations (GEE) for nested binomial outcomes with within-cluster treatments [88] as the best available model to estimate power for our primary outcome; such estimates are lacking for Hierarchical Linear Modeling (HLM) models. With 24 physicians and a miniumum of 9 patients per physician who are eligible for a clinical trial (i.e., a minimum of 216 patients for whom outcome measures can be obtained), a Type I error rate (α) of .05, and ICC of .05, and probability of success under the null hypotheses (*p*H_0_) of .25, we are well powered to detect *p*H_1_ of .35 (*b* = 0.48, odds-ratio = 1.61) with power > .99. Aim 2 is to examine whether patient race influences the effectiveness of both patient- and physician-focused interventions on our primary outcome; and we remain well powered to detect 2-way and 3-way interactions involving intervention condition and patient race. We will also examine effects of the interventions, and between-race differences in effects of the interventions on other binary or continuous secondary outcomes (e.g., trial offers, patients’ perceptions of patient-centeredness, trust in physician, etc.). For the continuous outcomes, we used block person-randomized trial module in Optimal Design [89] to estimate power. Considering each of the 24 physicians as “blocks” and assuming a minimum of 9 patients per physician, a Type I error rate (α) of .05, between-physicians variability in effect size (${\upsigma}_{\updelta}^2$) of .05, 5% of variance in outcomes due to physicians and a medium effect size (d) of .50, power to find effects exceeds .90. Our final objective (Aim 3) is to explore the extent to which patient-physician communication mediates the effects of the interventions on the outcomes. We will use Multi-level Structural Equation Modeling (MSEM) that control for patient-physician nesting to fit path analyses. The specific structure (i.e. direct and indirect paths of the models) will be guided by results from analyses conducted for our first and second aims. We therefore consider the MSEM exploratory in that we are the first researchers to examine these effects in this context. Thus, at this point we lack the specification of the model parameters needed to provide accurate estimates of power for this exploratory aim.

## Discussion

PACCT is highly significant in several ways. First, it can increase clinical trial participation rates of African-American and White men with prostate cancer, thus improving the generalizability of findings from these trials to a diverse patient population. Second, the research will provide empirical data regarding the theroretical mechanisms through which the interventions affect outcomes. Third, the design will provide descriptive information which is currently unavailable on the proportion of patients with prostate cancer who are eligible for a trial, are offered a trial, agree to participate, and/or enroll. Fourth, findings can inform the development of future interventions to improve trial enrollment of other underrepresented populations (e.g., Hispanic patients, older patients) and in other contexts. Fifth, multilevel interventions have the potential to achieve substantial and sustained change, and to produce effects that are at least additive and possibly multiplicative. Finally, this research directly addresses racial disparities in cancer care by improving access to high quality clinical care for African American men suffering the disproportionate burden of disparities in prostate and other cancers.

Although there are several strengths of the study, PACCT has some potential limitations. One of these is the focus on physicians, rather than on other members of the health care team, such as research nurses, who are clearly critical to enrolling patients in clinical trials. However, PACCT focuses on physicians because they make the final decision about the clinical appropriateness of a trial for a specific patient and are generally responsible for introducing the study to patients [90]. Also, patients consider physicians to be their primary and preferred source of information [71, 91–94]. Thus, physicians can present a primary barrier or facilitator to the enrollment process.

Another potential limitation is the focus on African Americans rather than on members of other minority groups. African Americans are the focus of this study primarily because members of this community bear the disproportionate burden of prostate and other cancers, as compared to White patients [95]. Increasing participation rates of African-American men with prostate cancer is particularly important because of the higher incidence, morbidity, and mortality rates among African-American men as compared to White men [96]. Additionally, the conceptual model and preliminary data upon which PACCT is based focus on research specific to African Americans. However, a strength of this study is that it will provide evidence for interventions and the mechanisms through which these interventions affect outcomes; this research can therefore inform interventions to benefit other minority communities in the future.

## Abbreviations

CIOMS: Council for International Organizations of Medical Sciences
GEE: General Estimating Equation
HLM: Hierarchical Linear Modeling
KCI: Karmanos Cancer Institute
MLM: Multilevel Model
MSEM: Multi-level Structural Equation Modeling
NCI: National Cancer Institute
PACCT: Partnering Around Cancer Clinical Trials
QPL: Question Prompt List
SKCCC: Sidney Kimmel Comprehensive Cancer Center
WHO: World Health Organization
WSU: Wayne State University
